# Seroprevalence of Antibodies to Avian Influenza A (H5) and A (H9) Viruses among Market Poultry Workers, Hanoi, Vietnam, 2001

**DOI:** 10.1371/journal.pone.0043948

**Published:** 2012-08-21

**Authors:** Timothy M. Uyeki, Doan C. Nguyen, Thomas Rowe, Xiuhua Lu, Jean Hu-Primmer, Lien P. Huynh, Nguyen L. K. Hang, Jacqueline M. Katz

**Affiliations:** 1 Influenza Division, National Center for Immunization and Respiratory Diseases, Centers for Disease Control and Prevention, Atlanta, Georgia, United States of America; 2 Department of Virology, National Institute of Hygiene and Epidemiology, Ministry of Health, Hanoi, Vietnam; University of Hong Kong, Hong Kong

## Abstract

**Background:**

The frequency of avian influenza A virus infections among poultry workers is not well understood.

**Methods:**

A seroprevalence study of market poultry workers and persons without occupational poultry exposure was conducted during 2001 in Hanoi, Vietnam. Sera were tested for avian influenza H5 and H9 antibodies by microneutralization and Western blot assays.

**Results:**

Seroprevalence of H5 and H9 antibodies was 4% and 3% in poultry workers and 1% and 3.5% in non-poultry workers, respectively.

**Conclusions:**

Seroprevalence of H5 and H9 antibodies was low among Hanoi market poultry workers in 2001, but can serve as a baseline for additional studies.

## Introduction

Human infections with highly pathogenic avian influenza (HPAI) A (H5N1) virus were first identified in Hong Kong during 1997 [1] and again in 2003 [2]. HPAI H5N1 viruses have evolved into multiple genetically and antigenically distinct clades and subclades, spreading among poultry in many countries. As of July 6, 2012, 15 countries had reported a total of 607 confirmed H5N1 human cases with 59% mortality since November 2003, including 123 cases with 61 deaths reported from Vietnam [3]. Vietnam is considered an endemic country where different clades and subclades of HPAI H5N1 viruses circulate among poultry [4]. Most human cases have resulted from sporadic avian-to-human transmission of H5N1 virus during direct or close contact with sick or dead poultry [5]. Visiting a poultry market has also been identified as a risk factor for human infection with H5N1 virus in Hong Kong and mainland China [6].

Sporadic human cases of low pathogenic avian influenza (LPAI) A (H9N2) virus infection, generally causing mild illness, have been reported in Hong Kong and mainland China since the late 1990s [7]. Although the source and risk factors for transmission to humans have often not been identified, LPAI H9N2 viruses have circulated widely among poultry for years and are considered enzootic in some Asian and Middle East countries [8]. The risk of infection with HPAI H5N1 or LPAI H9N2 viruses among persons working in live poultry markets where these viruses are prevalent among poultry is not well understood. In a 1997–98 cross-sectional study of poultry workers in Hong Kong, including live poultry market workers, the estimated age-adjusted seroprevalence of antibodies to H5N1 virus was 10% [9]. In this study, the odds of testing seropositive for H5N1 virus antibodies were 2.7 times greater in retail poultry workers compared to workers employed in wholesale/hatchery/farm/other poultry operations, while stratified analysis suggested that butchering poultry and exposure to poultry with >10% mortality were associated with H5 seropositivity [9]. Limited data exist on the risk of LPAI H9N2 virus infection among poultry workers. One study in southern China reported H5 and H9 antibody prevalence among retail poultry market workers to be 0.8% and 15.5%, respectively [10].

In 2001, before the spread of HPAI clade 1 and clade 2.3 H5N1 viruses in Vietnam, we conducted a study to assess the prevalence of avian influenza A viruses among live poultry in Hanoi markets [11]. That study detected several avian influenza A viruses, including HPAI H5N1 virus in specimens collected from healthy geese, and LPAI H5N2 and H9N3 viruses in ducks [11]. In 2001, HPAI H5N1 viruses were sporadically detected in poultry, and were not associated with poultry outbreaks in Vietnam until 2003 [12]. The first human cases of HPAI H5N1 virus infection identified in Vietnam occurred in late 2003 [13]. Here we report the results of an antibody seroprevalence study conducted during 2001 with the objective of determining if persons with occupational exposure to poultry at live poultry markets in Hanoi had evidence of HPAI or LPAI H5 or LPAI H9 virus infections.

## Materials and Methods

### Ethics Statement

The study protocol was approved by the institutional review boards of the Centers for Disease Control and Prevention (CDC), Atlanta, GA, and the National Institute of Hygiene and Epidemiology, Hanoi, Vietnam.

### Sample and Procedure

In October 2001, after obtaining signed, informed consent, adults aged ≥18 years were enrolled in the study. Convenience sampling was done among adult workers at 11 of the largest live poultry markets in Hanoi, Vietnam to enroll 200 participants based on an estimated seroprevalence of 10% for either H5N1 or H9N2 antibodies among poultry workers [9]. Controls were selected by convenience sampling of university students and public health staff in Hanoi to enroll 200 adults without occupational poultry exposure, with frequency matching to market poultry workers (MPWs) participants by gender. A questionnaire was administered by trained study staff to MPWs and controls to collect demographic information and data on potential risk factors for exposure to live or killed poultry and swine at work, and outside of work. A 5cc blood specimen was obtained from all study participants for determination of serum antibodies to avian influenza A (H5) and (H9) viruses. Data were entered into a database and analyzed by descriptive statistics using Epi-Info 2000 software.

### Serologic assays

Sera from participants were tested for antibodies to H5 and H9 viruses by microneutralization assay (MN) and confirmatory Western blot assay (WB) at CDC as previously described [14], [15]. H5 viruses tested were two viruses isolated from poultry specimens collected from the same live poultry markets in 2001 as in this study, A/Goose/Vietnam/113/2001 (Gs/VN/113, HPAI H5N1) and A/Duck/Vietnam/342/2001 (Dk/VN/342, LPAI H5N2) [11] and an HPAI clade 0 virus, A/Hong Kong/156/1997 (HK/156) [16], and an ancestral clade 1 virus, A/Hong Kong/213/2003 (HK/213) [17], isolated from fatal human cases in Hong Kong in 1997 and 2003, respectively. LPAI H9 viruses tested were A/Duck/Vietnam/339/2001 (Dk/VN/339, H9N3; H9 Korea lineage) isolated from a duck in Hanoi, Vietnam in 2001 [11], A/Hong Kong/1073/1999 (HK/1073, H9N2; G1 lineage), isolated from a child in Hong Kong in 1999 [18], and a reassortant H9N7 virus possessing the hemagglutinin (HA) of A/Chicken/Hong Kong/G9/1997 (Ck/G9, H9N2; Y280 lineage) and the neuraminidase (NA) from A/Equine/Prague/1/56 (H7N7) virus. The latter virus was used to exclude detection of cross-reactive anti-N2 antibody responses that may confound serologic results. Sera were also tested for antibodies to human influenza A viruses of the H1, H2, H3 subtypes for control purposes (data not shown). Ferret antisera raised against homologous viruses were used as positive control sera for the assays. Human antisera were tested at a starting dilution of 1∶10.

Sera positive by the MN assay for H5 or H9 antibodies were analyzed by WB using purified baculovirus-expressed recombinant H5 (HK/156) or H9 (HK/1073) HA produced by Protein Sciences (Meriden, CT, USA), as previously described [14]. Western blot was used as a confirmatory assay because of its relevance for testing antibody to both H5 and H9 subtypes.

To remove antibodies in sera known to cross-react with some H9 viruses, sera that were positive by MN and WB for antibodies to H9 viruses were adsorbed with a reassortant H2N7 virus possessing the HA of A/Japan/305/1957 (H2N2) virus and the NA from A/Equine/Prague/1/56 (H7N7) virus for individuals ≥33 years of age [19], and with a contemporary H3N2 virus A/Panama/2007/1999 for those <33 years who would not have been exposed to H2N2 viruses. We used the latter virus because of our unpublished observation that unexposed persons born in or after 1968 may exhibit cross-reactivity antibody to H9N2 viruses and that such cross-reactivity can be removed from sera by adsorption with contemporary H3N2 virus. Briefly, 100 micrograms of purified influenza virus was added to 50 microliters of sera and mixed. The samples were incubated for 2.5 hours at 4°C. Virus was removed by centrifugation (∼100,000g×45 minutes) and serum was adsorbed three times with 10 microliters of packed turkey red blood cells to remove any residual virus. Serum volume was adjusted to 100 microliters with phosphate buffered saline and samples were heat-inactivated for 30 minutes at 56°C. Removal of residual virus was verified by the lack of hemagglutination. Adsorbed sera were retested for neutralizing antibody to H9 viruses using the MN assay.

### Criteria for seropositivity

An individual was considered seropositive for H5N1 virus antibody if a MN titer of ≥40 (equivalent to a titer of ≥80 with a 1∶20 starting dilution per World Health Organization guidelines [20]) was obtained in duplicate tests, and confirmed by WB. For H9 viruses, sera that met the above criteria and achieved a MN titer of ≥40 following absorption with H3N2 and H2N7 viruses were considered positive for H9 antibody.

## Results

### Characteristics of study population

Controls (n = 200) were younger (median age 22 years versus 32 years), completed more years of higher education (89.9% completed secondary school versus 40.1%), had higher household income (76% had a median of ≥1 million Vietnamese Dong/month versus 66.5%), lived in Hanoi for a shorter period (median 11.1 years versus 21.1 years), and were less prevalent smokers (15.6% versus 23.5%), than MPWs (n = 200), respectively. Both MPWs and controls reported a median of 4 household members. The proportion of participants with live poultry inside the home (19% versus 20%) and with live poultry next to the home (28.5% versus 24.5%) were similar among MPWs and controls, respectively. The proportion with live pigs outside the home was higher among MPWs (35%) than controls (18.5%) [p = 0.0003]. Six percent of MPWs reported previous farm poultry work versus none among controls. MPWs reported working in live poultry markets a median of 5.5 years, range 1–40 years. Females had more experience (median 7 years) than male MPWs (median 3 years).

### Seroprevalence of H5 antibodies

Since the MN assay detects strain-specific antibody, we tested sera against multiple H5 viruses including HPAI H5N1 viruses representing those that infected humans at the time of serum collection [11]. Two male MPWs who worked at the same market were seropositive to Gs/VN/113, a HPAI H5N1 clade 0 virus isolated from a healthy goose at a different Hanoi market during 2001 (Table 1). Five female MPWs who worked at 4 different poultry markets were seropositive to two other HPAI H5N1 viruses [HK/156 (clade 0) and HK/213 (ancestral clade 1)]. One additional female MPW was seropositive against HK/213, and 2 NPWs were seropositive for both HK/213 and HK/156 (H5N1) viruses. Overall, the seroprevalence of antibodies against any HPAI H5N1 virus was 4% among MPWs versus 1% among controls (p = 0.1050) No participants were seropositive against Dk/VN/342 (H5N2), a LPAI virus isolated from a duck specimen at a Hanoi market in 2001 [11].

**Table 1:** Number and percent of live poultry market workers and non-poultry worker controls whose serum tested positive for neutralizing antibody against H5 viruses.

Virus used in Microneutralization assay	No. (%) seropositive for neutralizing antibody^a^		
	Poultry market workers (n = 200)	Control group (n = 200)	*p-value* *
A/Gs/VN/113/01 (H5N1)	2 (1.0)	0 (0)	0.4987
A/Dk/VN/342/01 (H5N2)	0 (0)	0 (0)	1.0000
A/HK/213/03 (H5N1)	6 (3.0)^b^	2 (1.0)^c^	0.2841
A/HK/156/97 (H5N1)	5 (2.5)^b^	2 (1.0)^c^	0.4490
Any H5 virus+	8 (4.0)	2 (1.0)	0.1050

a Sera were considered positive for antibody to an H5 virus if a titer of ≥40 was obtained in two independent assays and tested positive by Western immunoblotting for reactivity with purified H5 HA.

b Sera from 5 individuals in the poultry market worker group were seropositive for neutralizing antibody to HK/156/97 and HK/213/03 viruses.

c Sera from the same 2 individuals from the control group were seropositive for neutralizing antibody to HK/156/97 and HK/213/03 viruses.

* Fischer's Exact Test.

### Seroprevalence of LPAI H9 antibodies

No participants were seropositive to HK/1073 (H9N2; G1 lineage), (Table 2). One poultry worker was seropositive for antibodies against Gs/VN/339 (H9N3) isolated from a goose specimen at a Hanoi market in 2001 [11]. The seroprevalence of antibodies to the H9N7 reassortant derived from Ck/G9 was 2.5% in MPWs and 3.5% in controls. Overall, there was no difference in the seroprevalence of antibodies to any H9 virus among MPWs (3%) compared to controls (3.5%) [p = 1.000]. One market poultry worker tested seropositive for antibodies against both LPAI H9N2 (G9 lineage), and HPAI H5N1 viruses (HK/156) and (HK/213).

**Table 2:** Number and percent of live poultry market workers and non-poultry worker controls whose serum tested positive for neutralizing antibody against H9 viruses by age.^a^

Virus used in Microneutralization assay	No. (%) seropositive for neutralizing antibody^b^									
	Poultry market workers (n = 200)					Control group (n = 200)				
	<33 years (n = 104)		≥33 years (n = 96)		All ages	<33 years (n = 183)		≥33 years (n = 17)		All ages
	Non-adsorbed	Adsorbed^c^	Non-adsorbed	Adsorbed	Adsorbed	Non-adsorbed	Adsorbed	Non-adsorbed	Adsorbed	Adsorbed
A/Gs/VN/339/01 (H9N3)	1 (1)	0 (0)	9 (9)	1 (1)	1 (0.5)	1 (0.5)	0 (0)	6 (35)	0 (0)	0 (0)
A/HK/1073/99 (H9N2)	0 (0)	0 (0)	1 (1)	0 (0)	0 (0)	0 (0)	0 (0)	0 (0)	0 (0)	0 (0)
A/Ch/HK/G9/97 (H9N7)^d^	8 (8)	4 (4)	4 (4)	1 (1)	5 (2.5)	8 (4)	7 (4)	1 (6)	0 (0)	7 (3.5)*
Any H9 virus	8 (8)	4 (4)	14 (15)	2 (2)	6 (3)	9 (5)	7 (4)	7 (41)	0 (0)	7 (3.5)**

a Individuals were stratified by age to control for prior exposure to human influenza viruses. In 2001, individuals born in or before 1968 were ≥33 years of age and may have experienced infection with H2N2 and H3N2 viruses while individuals born after 1968 were <33 years of age would not have been exposed to H2N2 viruses.

b Non-adsorbed sera were considered to be preliminarily positive for antibody to an H9 virus if a titer of ≥40 was obtained in two independent assays and tested positive by Western immunoblotting for reactivity with purified recombinant H9 HA.

c Sera were adsorbed with human influenza viruses depending on the age of the individual. Sera from individuals <33 years of age were adsorbed with the contemporary human H3N2 virus A/Panama/2007/99; sera from individuals ≥33 years of age were adsorbed with the H2N7 reassortant virus.

d H9N7 virus is a reassortant possessing the hemagglutinin (HA) of A/Chicken/Hong Kong/G9/1997 virus (H9N2) and the neuraminidase (NA) from A/Equine/Prague/1/56 (H7N7) virus.

* p = 0.7710 Fischer's Exact Text.

** p = 1.000 Fischer's Exact Text.

## Discussion

While seroprevalence of HPAI H5N1 virus antibodies was low, we were able to detect evidence of human infections with HPAI H5N1 virus in Hanoi, Vietnam during 2001, after the 1997 outbreak of clade 0 HPAI H5N1 cases in Hong Kong and before the emergence of clade 1 HPAI H5N1 cases in 2003 [1], [2]. Overall, 8 Hanoi MPWs had antibodies against one or more HPAI H5N1 viruses tested. HPAI H5N1 virus antibodies were detected at a higher frequency among MPWs than among controls, although this was not significantly different. Two non-poultry worker controls were seropositive for antibodies to both HPAI H5N1 viruses isolated in Hong Kong in 1997 and 2003, highlighting the importance of non-occupational exposure to poultry as a risk factor for HPAI H5N1 virus infection [5], [6]. Serologic evidence of human infection with an HPAI H5N1 virus isolated from healthy geese in two Hanoi poultry markets at the time of the study was found in 2 MPWs, but not in controls.

The 4% seroprevalence of clade 0 HPAI H5N1 virus antibodies in 2001 among MPWs was slightly higher than reported for clade 0 or clade 1 HPAI H5N1 virus antibodies among a similar population in southern China during 2007–08 (0.8%) [10], but less than the estimated 10% seroprevalence of clade 0 HPAI H5N1 antibodies among Hong Kong poultry workers during 1997–98 [9]. Since the kinetics of the HPAI H5N1 virus antibody response in infected humans with asymptomatic or clinically mild illness suggest that antibody titers decline below the WHO seropositive threshold [18] over 6–12 months following exposure [21], the seropositives we identified most likely indicate relatively recent HPAI H5N1 virus infection in relationship to sera collection in October 2001. It is possible that other HPAI H5N1 virus infections could have been missed in the study participants if a limited antibody titer response occurred or if the titer had declined below the threshold for a seropositive. Nevertheless, these studies and others indicate the potential for HPAI H5N1 virus to cause asymptomatic infection or mild illness in adults. We also identified a similar low seroprevalence of antibodies to LPAI H9N2 virus among MPWs and non-poultry worker controls in Hanoi, suggesting that occupational exposure to poultry was not a major risk factor for LPAI H9N2 virus infection among the study population in 2001.

These findings are limited by the cross-sectional study design, and the low seroprevalence of H5 or H9 antibodies, for which the study was underpowered to detect significant differences to identify risk factors for human infection with HPAI H5N1 or LPAI H9N2 viruses. Since both study populations were selected by convenience sampling, and the MPWs were older than controls, our findings may not be applicable to other populations in Vietnam. A particular strength of this study is the extensive laboratory methods performed to ensure reliability of the serological results, including performing adsorption with H2N7 and H3N2 viruses to minimize detection of H9 seropositivity due to cross-reactivity with antibodies to human influenza A viruses. In one H9N2 vaccine study, individuals born before 1969 exhibited pre-vaccination neutralizing titers against H9N2 (G1) [19]. In another H9N2 vaccine study that was conducted in the U.S., baseline pre-vaccination H9N2 (G1 and G9 lineages) virus neutralizing antibody titers were higher in persons aged 44–59 years compared to 18–38 years, whereas titers detected by hemagglutination-inhibition assay were higher to the G9 lineage virus compared with the G1 lineage virus in both age groups [22]. Following adsorption with H2N7 virus, we observed a reduction in the prevalence of individuals with neutralizing antibodies to H9N2 viruses in persons aged ≥33 years. We also observed a reduction in the prevalence of neutralizing antibodies to H9N2 viruses in persons aged <33 years after adsorption with H3N2 virus. Taken together with the previous studies, these findings suggest that there may be age-, virus-, and assay-specific differences in cross-reactivity when detecting antibodies to H9N2 virus, and that additional serologic investigations are needed. Of note, other published H9N2 virus antibody seroprevalence studies either did not address or did not perform additional laboratory tests to reduce or eliminate potential cross-reactivity with other human influenza A virus subtype antibodies which may limit the interpretation of their results [10], [23]–[29].

The findings from this study can serve as a baseline for additional serologic studies to assess avian-to-human transmission of HPAI H5N1 and LPAI H9N2 viruses as the prevalence of these evolving viruses has increased substantially among poultry in northern Vietnam since 2001.
